# Ethanol Mediated Inhibition of Synaptic Vesicle Recycling at Amygdala Glutamate Synapses Is Dependent upon Munc13-2

**DOI:** 10.3389/fnins.2017.00424

**Published:** 2017-07-21

**Authors:** Dominic A. Gioia, Nancy Alexander, Brian A. McCool

**Affiliations:** Department of Physiology and Pharmacology, Wake Forest School of Medicine Winston Salem, NC, United States

**Keywords:** presynaptic, lateral/basolateral amygdala, medial prefrontal cortex, vesicle priming, shRNA

## Abstract

Chronic exposure to alcohol produces adaptations within the basolateral amygdala (BLA) that are associated with the development of anxiety-like behaviors during withdrawal. In part, these adaptations are mediated by plasticity in glutamatergic synapses occurring through an AMPA receptor mediated form of post-synaptic facilitation in addition to a unique form of presynaptic facilitation. In comparison to the post-synaptic compartment, relatively less is understood about the mechanisms involved in the acute and chronic effects of ethanol in the presynaptic terminal. Previous research has demonstrated that glutamatergic terminals in the mouse BLA are sensitive to ethanol mediated inhibition of synaptic vesicle recycling in a strain-dependent fashion. Importantly, the strain-dependent differences in presynaptic ethanol sensitivity are in accordance with known strain-dependent differences in ethanol/anxiety interactions. In the present study, we have used a short-hairpin RNA to knockdown the expression of the presynaptic Munc13-2 protein in C57BL/6J mice, whose BLA glutamate terminals are normally ethanol-insensitive. We injected this shRNA, or a scrambled control virus, into the medial prefrontal cortex (mPFC) which sends dense projections to the BLA. Accordingly, this knockdown strategy reduces the expression of the Munc13-2 isoform in mPFC terminals within the BLA and alters presynaptic terminal function in C57BL/6J mice in a manner that phenocopies DBA/2J glutamate terminals which are normally ethanol-sensitive. Here, we provide evidence that manipulation of this single protein, Munc13-2, renders C57BL/6J terminals sensitive to ethanol mediated inhibition of synaptic vesicle recycling and post-tetanic potentiation. Furthermore, we found that this ethanol inhibition was dose dependent. Considering the important role of Munc13 proteins in synaptic plasticity, this study potentially identifies a molecular mechanism regulating the acute presynaptic effects of ethanol to the long lasting adaptations in the BLA that occur during chronic ethanol exposure.

## Introduction

Alcohol use disorders (AUDs) are frequently comorbid with anxiety related disorders including generalized anxiety disorder, panic disorder, and post-traumatic stress disorder (Regier et al., 1990). The co-occurrence of these disorders presents a substantial barrier to efficacious treatment, as high levels of anxiety are major factors contributing to relapse (Dawson et al., 2007; Simioni et al., 2012). In order to provide effective treatments for people with comorbid anxiety and AUDs, it will be important to further understand the mechanisms involved in alcohol-induced adaptations in anxiety related brain regions.

The basolateral amygdala (BLA) is an important brain region that is thought to contribute to both the anxiolytic effects of ethanol intoxication as well as the anxiogenic effects of withdrawal from chronic ethanol exposure (Läck et al., 2007). We have previously shown that chronic ethanol exposure induces a presynaptic form of plasticity in the medial glutamatergic inputs to the BLA (Christian et al., 2013) and that microinjection of glutamatergic antagonists into the BLA prevents the expression of withdrawal related anxiety (Läck et al., 2007). Together, these studies suggested that ethanol related plasticity in presynaptic medial inputs to the BLA may drive the expression of anxiety-like behavior during withdrawal. Although, the mechanisms involved in this plasticity are currently unknown, they may provide novel therapeutic targets for people with comorbid alcohol use and anxiety disorders.

The neural circuitry controlling anxiety-like behavior is well-conserved across mammalian species (Janak and Tye, 2015). Accordingly, animal models can be useful for precise investigation into the neurobiological mechanisms controlling interactions between alcohol and anxiety. Along these lines, inbred mouse strains are particularly powerful models which allow a detailed understanding of how genotypes contribute to vulnerability or resistance to ethanol-related anxiety. For example, DBA/2J (D2) mice are sensitive to both the anxiolytic effects of acute ethanol as well as the anxiogenic effects of chronic ethanol exposure while the C57BL/6J (B6) mice are resistant to both (McCool and Chappell, 2015). Rodent models are also highly amenable to studies focused on cause-effect relationships to be made between genotype and neural circuitry. We recently examined basal glutamatergic transmission in lateral/basolateral amygdala neurons in “vulnerable” (D2) and “resistant” (B6) mouse strains. In that study, we showed that neurons from D2 mice expressed a more robust presynaptic phenotype relative to neurons in B6 mice which was regulated by strain-dependent differences in the expression of the presynaptic protein, Munc13-2 (Gioia et al., 2016). In that study, a shRNA was used to knockdown the expression of Munc13-2 in the mPFC, which sends direct monosynaptic projections through medial inputs to the BLA. This manipulation produced a “D2-like” presynaptic phenotype in the BLA of B6 mice. Considering that medial projections to the BLA are known to undergo plasticity during chronic ethanol exposure along with the strain-dependent differences in withdrawal anxiety vulnerabilities, we hypothesized that strain-dependent differences in Munc13-2 expression may regulate presynaptic effects of ethanol.

The Munc13 gene family expresses 4 different isoforms, Munc13-(1-4), which are essential for synaptic vesicle priming, vesicle recycling, and long term plasticity (Varoqueaux et al., 2002; Yang and Calakos, 2011). Cortical regions like the BLA express both the Munc13-1 and Munc13-2 isoforms while the other isoforms are expressed elsewhere in the brain and throughout the body (Augustin et al., 1999; Gioia et al., 2016). Munc13-1 and Munc13-2 have conserved C-terminal regions but highly divergent N-terminal regions that alter their function within the terminal. Munc13-1 contains a C2A domain that interacts with RIM1 proteins to position primed vesicles in close proximity to voltage gated calcium channels at the presynaptic active zone (Betz et al., 2001; Andrews-Zwilling et al., 2006), while the brain specific isoform of Munc13-2 lacks this domain (Shin et al., 2010). These differences between isoforms have been shown to alter basal release characteristics as well as short and long term plasticity. For example, Munc13-2 acts as a negative modulator of vesicle release probability due to competition over SNARE complex proteins with Munc13-1 within the same terminals (Chen et al., 2013). Consistent with this, we found that B6 mice express a higher ratio of Munc13-2 to Munc13-1 relative to the D2 strain, and that knockdown of Munc13-2 expression facilitated glutamate release in B6 mice (Gioia et al., 2016).

More recently, we found that D2 glutamatergic synapses were more sensitive to ethanol-mediated inhibition of synaptic function relative to B6 synapses (Gioia and McCool, 2017). Specifically, we found that, while there were no effects of alcohol on the size of the readily releasable pool of vesicles (RRP) in either strain, ethanol specifically inhibited synaptic vesicle recycling in a strain-dependent fashion. Importantly, Munc13-1 proteins, critically important mediators of this activity, encode an ethanol binding site (Das et al., 2013; Kabachinski et al., 2014; Martin, 2015) which can dramatically alter its function. Together, these studies suggest that differences in the expression/function of distinct Munc13 isoforms may contribute to differences in ethanol inhibition of synaptic vesicle recycling. In this study, we directly tested this hypothesis by using an established short-hairpin RNA approach to knock down the expression of the Munc13-2 isoform within glutamate projections to the BLA of B6 mice. We measure the impact of this knockdown on ethanol modulation of synaptic vesicle recycling, and a Munc13-dependent form of short-term plasticity.

## Materials and methods

### Animals

Five week old male C57BL/6J mice were obtained from The Jackson Laboratories (Bar Harbor, ME, cat. #000664) and given access to food and water *ad libitum*. Mice were group housed for 1–2 weeks in a facility maintained by institutional animal resource personnel with housing conditions consistent with the NIH Guidelines for the Care and Use of Laboratory Animals (68–74°F, 30–70% relative humidity) prior to surgery. All experimental procedures on animals were reviewed and approved by the WFSM Animal Care and Use Committee.

### Western analysis

Total protein was prepared from GFP-positive regions of the prefrontal cortex (see below) using standard methods (Gioia et al., 2016) from to different cohorts. 10 μg total protein was fractionated on a 4–20% sodium dodecyl sulfate precast polyacrylamide gel (Bio-Rad, catalog # 567-1094) and transferred to a nitrocellulose membrane (Invitrogen, catalog # IB301001). Expression was detected using commercially available antibodies and standardized to total protein levels determined from Image Lab software (Bio-Rad) analysis of reversible total protein stain (Thermo Scientific, catalog #24580) in each gel lane. Standardized values are reported in arbitrary units for each protein. The specificity of individual antibodies was indicated by the detection of immunoreactive bands at the appropriate molecular weight and by blocking the immunoreaction with antigenic peptides: Munc13-1 (Synaptic Systems, catalog #126 102, 1:1,000), ubMunc13-2 (Thermo Scientific, catalog # PA5-22159, 1:5,000), Rab3a (Synaptic Systems, catalog #107 111, 1:1,000), RIM1α (Synaptic Systems, catalog # 140 023, 1:3,000), SNAP-25 (Synaptic Systems, catalog #111 002, 1:5,000), Syntaxin 1 (Millipore, catalog #AB5820, 1:10,000), and VAMP1 (AbCam, catalog #ab3346, 1:5,000). Immunoreactive bands were detected using standard chemiluminescent detection with a species-appropriate secondary antibody.

### Electrophysiology methods

#### Slice preparation

Animals were anesthetized with isoflurane and decapitated. Brains were quickly removed and incubated in an ice-cold sucrose modified artificial cerebral spinal fluid (aCSF) containing (in mM): 180 sucrose, 30 NaCl, 4.5 KCl, 1 MgCl2·6H2O, 26 NaHCO3, 1.2 NaH2PO4, 0.10 ketamine, and 10 glucose, equilibrated with 95% O_2_ and 5% CO_2_. Coronal slices containing the BLA were obtained (300 μm) using a VT1200 S vibrating blade microtome (Leica, Buffalo Grove, IL) and were incubated for at least 1 h in room temperature (~25°C), oxygenated standard aCSF containing (in mM): 126 NaCl, 3 KCl, 1.25 NaH2PO4, 2 MgSO4, 2 CaCl2, 26 NaHCO3, and 10 glucose, before initiation of recordings.

#### Whole cell patch clamp recordings

BLA slices were transferred to a submersion-type recording chamber and perfused with room temperature (~25°C) aCSF (2.0 ml/min) for whole-cell voltage clamp recordings similar to previously published reports (Christian et al., 2013). Data were acquired via Axopatch 700B (Molecular Devices, Foster City, CA) and analyzed offline via pClamp software (Molecular Devices, version 10.5). Inclusion criteria for presumptive principal neurons included high membrane capacitance (>100 pF) and low access resistance in the whole-cell configuration (<20 MΩ, Washburn and Moises, 1992). Cells in which access resistance or capacitance changed ≥20% during the record or with changes in resting membrane currents ≥100 pA were excluded from analysis. Glutamatergic responses were pharmacologically isolated using 100 μM picrotoxin (a GABA_*A*_ antagonist) in the bath aCSF and were recorded with electrodes filled with an internal solution containing (in mM): 145 Cs-gluconate, 10 EGTA, 5 NaCl, 1 MgCl_2_, 10 HEPES, 0.4 QX314, 1 CaCl_2_-2H_2_O, 4 Mg-ATP, and 0.4 Na_3_-GTP. Osmolarity of internal solution was corrected to ~285 mOsm with sucrose and pH was adjusted to ~7.25 with D-Gluconic acid. Synaptic responses were electrically evoked using concentric bipolar stimulating electrodes (FHC Inc, Bowdoin, ME, cat. #CBAEB75) placed within the stria terminalis.

#### 40 Hz stimulation protocol

Medial inputs to the BLA were stimulated at 40 Hz for 100 pulses, and then ethanol was bath applied to the recording chamber for 10 min followed by an additional 40 Hz 100 pulse train. Importantly, this stimulation protocol allows for the measurement of the size of both the readily releasable pool of vesicles, reflected by responses early in the train, and vesicle recycling capacity, reflected by responses later in the train. RRP was calculated by constructing a cumulative amplitude plot, obtained during 40 Hz stimulation, and performing a linear regression through the last 10 data points. The value of the y-intercept is then representative of the size of the RRP, while subtraction of the RRP from the total cumulative amplitude provides information on synaptic vesicle recycling rates (Schneggenburger et al., 2002; Gioia et al., 2016). We used electrical stimulation of the medial inputs to the BLA because we could not reliably elicit light evoked responses at frequencies >2 Hz which are required to evaluate different synaptic vesicle pools. However, we have previously demonstrated that this knockdown strategy effectively alters electrically evoked release probability through medial stimulation, thus justifying the use of electrical stimulation in these experiments.

#### Recovery of the readily releasable pool (RRP) and post-tetanic potentiation (PTP)

For these experiments, an initial 40 Hz/100 stimulus train was followed by a second, identical train using inter-train intervals of 1, 5, or 10 s. This allowed us to calculate the amount that the readily releasable pool recovered from the first train over a 1–10 s period. We used an area-under-the-curve calculation with the first five stimulations from each train to provide a measure of the total charge representing the readily releasable pool. Following this second train, ethanol was bath applied to the recording chamber for 10 min and this two train stimulation was repeated. Post-tetanic potentiation was measured as the amplitude of the first response in the second stimulus train divided by the amplitude of the first response in the first stimulus train and was expressed as a percent.

### Drug preparation

The GABA_A_ receptor antagonist picrotoxin (Tocris/Bio-Techne, Minneapolis, MN, cat. #1128) was prepared daily and dissolved in DMSO before adding to the recording aCSF. Ethanol (Warner-Graham Co., Cockeysville MD, 200 proof/non-denatured) was diluted with aCSF to the desired concentration and applied directly to the recording chamber via calibrated syringe pumps.

### Surgery

Mice were kept under continuous isoflurane anesthesia (4% for induction, 1.5% for maintenance) throughout the surgery which consisted of bilateral injection (1 μl over 10 min per side) of an AAV containing shRNA targeted to Munc13-2 (AAV5-GFP-U6-mUNC13B-shRNA, Vector Biolabs, Malvern PA, custom synthesis) or a scrambled control vector (AAV5-GFP-U6-scrmb-shRNA, Vector Biolabs, cat. #7040). Mice were given 5 mg/kg s.c. ketoprofen at the conclusion of the surgery for pain management. The medial prefrontal cortex was targeted using a Neurostar Stereodrive (Neurostar, Tübengen, Germany) and the coordinates 1.78 AP, ±0.33 ML, 2.35 DV relative to bregma. Mice were pair housed following surgery and allowed 4–6 weeks to recover for virus expression prior to *in vitro* experiments. Injection sites were confirmed using fluorescence microscopy *post-mortem*. The mPFC was chosen for the injection site because it sends dense monosynaptic projections through the medial aspects of the BLA directly onto local pyramidal cells (Gioia et al., 2016). Accordingly, this strategy allows us to manipulate presynaptic terminals in a pathway that is thought to be important for fear conditioning, alcohol related plasticity, and the expression of withdrawal related anxiety.

### Statistics

All graphs are plotted as mean ± SEM of each group. Primary statistical analyses were conducted using two-way ANOVA, one-way ANOVA, or *t*-tests (GraphPad, GraphPad Software Inc, La Jolla, CA) according to the experimental design as described, with post-tests as appropriate. The number of cells per group in each experiment is listed after each *p*-value.

## Results

We have previously demonstrated that microinjection of a Munc13-2 shRNA into the mPFC reduces Munc13-2 expression in both the mPFC and the BLA. This manipulation also reduces release probability at mPFC glutamatergic terminals and alters the frequency, but not amplitude, of spontaneous excitatory post-synaptic currents recorded from principal neurons in the BLA (Gioia et al., 2016). Thus, the manipulation of the levels of a single SNARE-associated protein in B6 mice produced a synaptic phenotype that mimics the phenotype of an unrelated mouse strain, DBA/2J. We also recently showed acute ethanol inhibition of synaptic vesicle recycling during a 40 Hz stimulation was both mouse strain- and frequency-dependent (Gioia and McCool, 2017). In order to determine if Munc13-2 isoforms were protective against this effect we injected the same shRNA Munc13-2 virus, or a scrambled control virus, into the mPFC of B6 mice and examined ethanol sensitivity during the delivery of a 100 pulse 40 Hz train.

We performed western analysis of total mPFC protein from mice injected with either scrambled control or Munc13-2 shRNA. Munc13-2 levels were significantly decreased by the shRNA (CON—0.87 ± 0.04, *n* = 7; shRNA—0.66 ± 0.08, *n* = 5; t = 2.778, *p* < 0.05, *t*-test) while levels of Munc13-1 were not altered in the same samples (CON—0.60 ± 0.02 A.U.; shRNA—0.58 ± 0.04; t = 0.293, *p* > 0.05, *t*-test). We also surveyed SNARE- and vesicular-priming proteins in these samples and found significant decreases in protein levels for SNAP-25 (CON—0.86 ± 0.03; shRNA—0.69 ± 0.08; t = 2.413, *p* < 0.05) and RIM1α (CON—1.15 ± 0.04; shRNA—0.89 ± 0.09; t = 2.712, *p* < 0.05). However, the knockdown did not disrupt the expression of Syntaxin 1 (CON—0.41 ± 0.03; shRNA—0.44 ± 0.03; t = 0.607, t = 0.607, *p* > 0.05), Rab3a (CON—0.14 ± 0.01; shRNA—0.15 ± 0.01; t = 0.416, *p* > 0.05), or VAMP1 (CON—1.18 ± 0.06; shRNA—1.14 ± 0.06; t = 0.435, *p* > 0.05). These data confirm previous results (Gioia et al., 2016) showing the Munc13-2 shRNA significantly reduces levels of the Munc13-2 protein and also indicate that, while the knockdown is selective with respect to Munc13 proteins in mPFC, there also appears to be a down-regulation in protein levels for the Munc13-binding protein Rim1α and the SNARE complex protein SNAP25.

### Ethanol inhibition of synaptic vesicle recycling is dependent on Munc13-2

**Figure 1:**
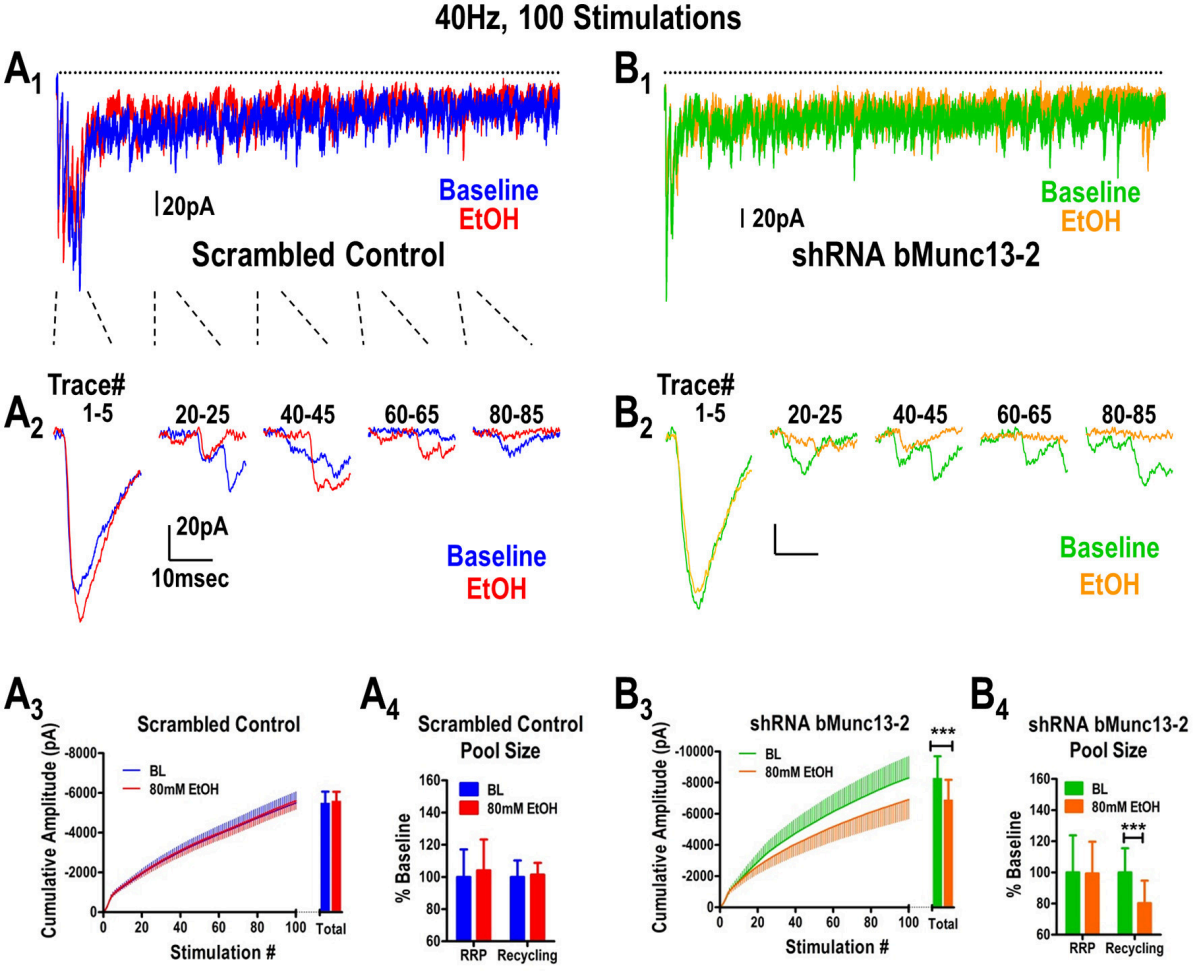
Ethanol inhibits glutamate synaptic vesicle recycling in a Munc13-dependent manner. **(A)** Ethanol does not inhibit BLA synaptic responses to a 40 Hz, 100 stimulus train delivered to the stria terminalis of B6 mice expressing scrambled shRNA control virus in mPFC inputs. Baseline traces (no ethanol) are indicated in blue while traces recorded in the presence of 80 mM ethanol are indicated in red **(A_1_,A_2_)**. All responses to the entire 40 Hz stimulus (dots above the traces) are shown in **(A_1_)** while individual responses corresponding to various sections of the stimulus train are illustrated in **(A_2_)**. A cumulative amplitude plot obtained during the 40 Hz stimulation **(A_3_)** shows that ethanol did not alter any portion of the stimulus train. Responses to the stimulus train can be used to quantify relative contributions by the readily releasable pool (RRP) and recycling pool of synaptic vesicles (see Methods). Neither the RRP nor the recycling pool contributions **(A_4_)** were affected by ethanol. **(B)** Expression of Munc13-2 in the mPFC was knocked down by microinjection of a AAV-viral construct expressing a shRNA to bMunc13-2. mPFC provides robust glutamatergic input to the BLA via the stria terminalis (Gioia et al., 2016). Representations of the entire 40 Hz train **(B_1_)** and individual traces within the train **(B_2_)** are illustrated using the same notations as in **(A)**. Munc13-2 knockdown significantly increased ethanol inhibition of both the cumulative response amplitude measured across the entire stimulus train (**B_3_**, paired *t*-test, ^***^p < 0.001) as well as the size of the recycling vesicle pool (**B_4_**, paired t-test, ^***^p < 0.001) but, importantly, not the size of the RRP (paired *t*-test, *p* = 0.93).

Using a 40 Hz stimulation protocol (Figures 1 A_1_, B_1_), we can easily separate synaptic responses mediated by the readily releasable pool of vesicles—represented by the few responses in the train—from those mediated by recycling synaptic vesicles. These later responses are frequently characterized by smaller amplitudes and greater onset latencies (i.e., more asynchronous) and a higher frequency of synaptic “failures” (i.e., no response to stimulus, Figures 1A_2_, B_2_). For example, despite initial response amplitudes of −95 ± 13pA in all control BLA cells (*n* = 14), 25 ± 4% of the stimulations during a 40 Hz train resulted in “failures” while a much greater percentage of the responses had latencies at least 25% greater than the initial response. This release phenotype during high-frequency stimulation is entirely consistent with many previous studies examining synaptic vesicle depletion (Hagler and Goda, 2001; Gioia and McCool, 2017).

In mice injected with the scrambled control virus, ethanol did not alter either the cumulative amplitude calculated from the 40 Hz stimulus (Figure 1A_3_, paired *t*-test, *p* = 0.69) or the sizes of the readily releasable (*t*-test, paired *p* = 0.71) and recycling vesicle pools (Figure 1A_4_, paired *t*-test, *p* = 0.80). This is consistent with our previous observations in control B6 mice (Gioia and McCool, 2017). However, in Munc13-2 knockdown mice (Figure 1B_3_, *n* = 15), we found that 80 mM ethanol significantly inhibited the total cumulative amplitude obtained during 40 Hz stimulation (paired *t*-test, *p* < 0.001). This was primarily due to inhibition of vesicle recycling since 80 mM ethanol did not alter the size of the RRP in the Munc13-2 knockdown (Figure 1B_4_, paired *t*-test, *p* = 0.93) but significantly inhibited the size of the recycling pool of synaptic vesicles (paired *t*-test, *p* < 0.001). This ethanol inhibition in the Munc13-2 knockdown animals is qualitatively similar to the ethanol inhibition of vesicle recycling observed in mouse strains like DBA/2J (Gioia and McCool, 2017) which have lower Munc13-2 levels (Gioia et al., 2016). However, Munc13-2 knockdown did not significantly alter the number of synaptic “failures” that occurred during the 40 Hz stimulation (baseline—22.8 ± 4.6 failures vs. EtOH—26.3 ± 5.6 failures, paired *t*-test, *p*~0.1). These data indicate that Munc13-2 levels dictate presynaptic ethanol sensitivity through inhibition of synaptic vesicle recycling.

### Ethanol inhibition of synaptic vesicle recycling in Munc13-2 knockdown animals is dose dependent

We then examined the concentration-dependent effects of this inhibition by repeating experiments in 60, 40, 10 and 0 mM ethanol. In control animals, no concentration of ethanol was able to significantly alter either the total cumulative amplitude of the 40 Hz train (one-way ANOVA across doses, *p*~0.39, not shown) or the size of the recycling vesicle pool (Figure 3A, one-way ANOVA, p~0.51) relative to the no-ethanol condition. On the other hand, in Munc13-2 knockdown animals, ethanol significantly inhibited both the total cumulative amplitude (one-way ANOVA, *p* < 0.001, Dunnett's multiple comparison post-test, 0 vs. 40 mM—*p* < 0.05, 0 vs. 60 mM—*p* < 0.001, 0 vs. 80 mM—*p* < 0.001) as well as the size of the recycling pool (Figure 3B, one-way ANOVA, *p* < 0.001). These data strongly suggest that ethanol inhibition of the recycling pool in Munc13-2 animals is concentration-dependent.

**Figure 2:**
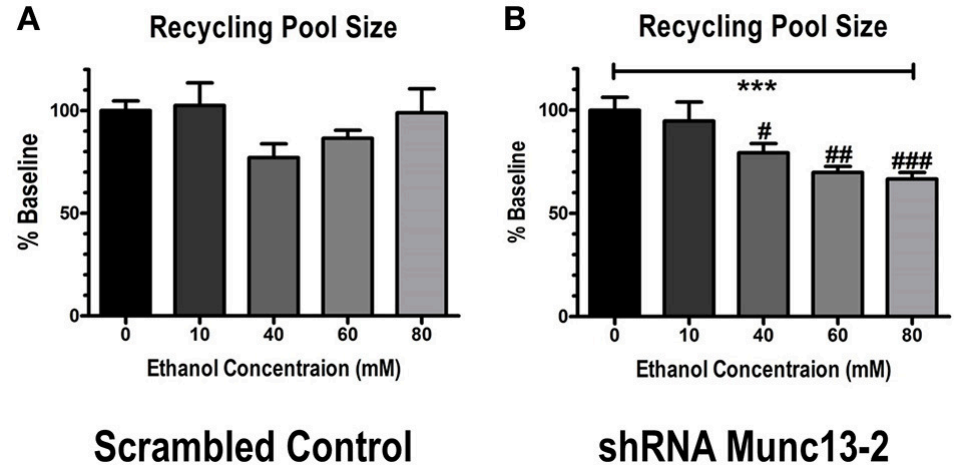
Munc13-2-dependent ethanol inhibition of the recycling pool of synaptic vesicles is concentration-dependent. **(A)** Concentration-response relationship for inhibition of synaptic vesicle recycling pool size in BLA neurons from B6 animals injected with scrambled control virus. Ethanol did not significantly inhibit recycling pool size (one-way ANOVA, *p* = 0.51). **(B)** Concentration-response relationship in BLA neurons injected with virus expressing shRNA against bMunc13-2. Ethanol inhibition of recycling pool size was both significant (one-way ANOVA, ^***^p < 0.001) and concentration-dependent (Dunnett's multiple comparison post-test, #*p* < 0.05, ##*p* < 0.01, ###*p* < 0.001 vs. 0 mM ethanol).

**Figure 3:**
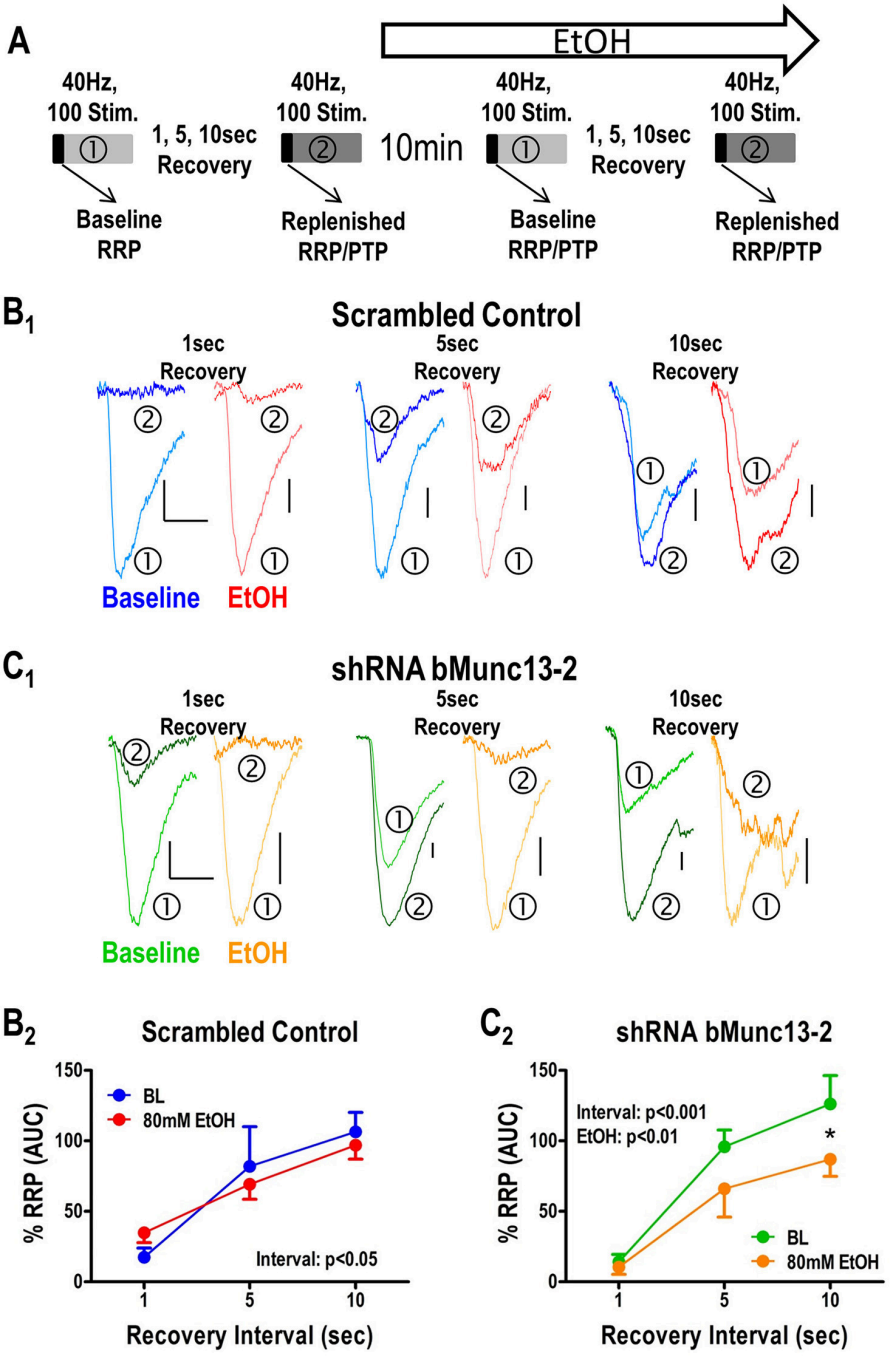
Recovery of the RRP following tetanic stimulation is Munc13-2 dependent and ethanol sensitive. **(A)** Illustration of the stimulation protocol used to measure RRP recovery. To measure recovery of the RRP, a single 40 Hz, 100 stimulus train (①) was followed by a second 40 Hz, 100 stimulus train (②) with increasing inter-train intervals of 1, 5, and 10 s. The effects of ethanol on recovery were measured 10 min after acute application to the slice. **(B)** Effects of ethanol on RRP recovery measured in BLA neurons from animals injected with the scrambled control virus. Representative traces corresponding to the first response in each train are shown **(B_1_)** with baseline and ethanol condition represented by blue and red traces, respectively, for each recovery interval. Area-under-the-curve measures **(B_2_)** for the first five responses were used as a proxy for the RRP. There was a significant main effect of recovery interval (two-way ANOVA, *p* < 0.05) but no significant effect of ethanol and no interaction between these main factors. **(C)** Effects of ethanol on RRP recovery measured in BLA neurons from animals injected with the virus expression shRNA against bMunc13-2. As in **(B)**, representative traces from the first response of each train across the various recovery intervals are shown **(C_1_)**. There were significant main effects for both recovery interval (two-way ANOVA, *p* < 0.001) and ethanol (*p* < 0.01) with recovery at the 10 s interval being significantly inhibited by ethanol relative to baseline (Bonferroni's post-test, ^*^p < 0.05).

### Ethanol inhibition of both releasable pool recovery and post-tetanic potentiation following high frequency stimulation is Munc13-2 dependent

Lastly, the recovery of the readily releasable pool of synaptic vesicles following periods of intense synaptic activity requires robust synaptic vesicle recycling. Accordingly, the mechanisms controlling synaptic vesicle recycling, like the levels of Munc13-2 (Gioia et al., 2016) and acute ethanol exposure (Gioia and McCool, 2017), should also modulate the recovery of the readily releasable synaptic vesicle pool. To measure this process, we modified the 40 Hz/100 stimulus protocol to consist of two stimulus trains separated by increasing inter-train intervals and tested the effects of acute ethanol (80 mM) on the interval-dependent recovery of the RRP (Figure 3A). In cells from animals injected with the scrambled control virus (Figure 3B_1_), the recovery of the RRP was both interval-dependent and insensitive to acute ethanol. In the absence of acute ethanol (Figure 3B_1_, blue traces), comparison of the first responses in each train showed that there was very little recovery during a 1 s interval with increasing recovery as intervals increased, as expected with time-dependent recovery. However, post-tetanic potentiation (PTP) was evident following a 10 s interval; as such, the first responses in the second train were larger than the first response in the first train at this interval this interval. Importantly, acute 80 mM ethanol did not appear to significantly alter this recovery process in the control cells (Figure 3B_1_, red traces). To more precisely quantify the recovery of the readily releasable pool, we compared the area under the curve (AUC) of the first five pulses in each train this was deemed a sufficient measure of the charge carried by the RRP. We found that this AUC was likewise significantly increased during increasing inter-train intervals, indicative of normal vesicle recycling, but was insensitive to acute ethanol (Figure 3B_2_, two-Way ANOVA, no interaction, *p* = 0.31, main effect of recovery interval, *p* < 0.01, no main effect of ethanol, *p* = 0.46).

**Figure 4:**
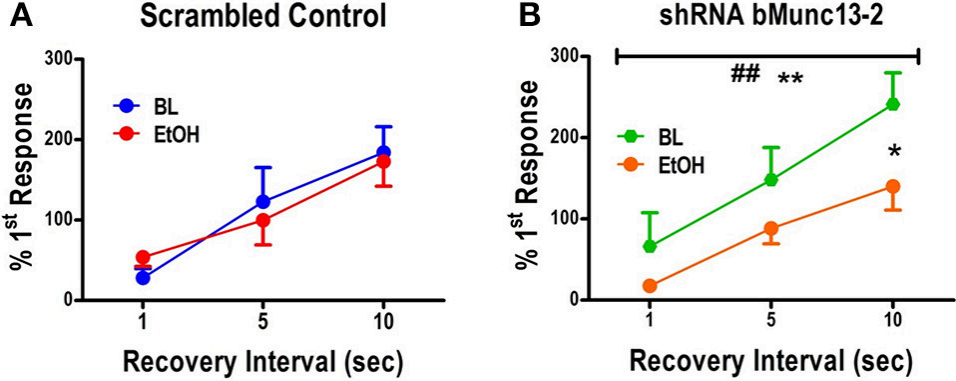
Post-tetanic potentiation is Munc13-2 dependent and ethanol sensitive. **(A)** Post-tetanic potentiation expressed as the percent amplitude of the first response in the second 40 Hz, 100 stimulus train relative to the first response in the first train. In BLA neurons from B6 mice injected with scrambled control virus, increasing recovery intervals significantly increased the initial second train response amplitude relative to the first train percent first response values (two-way ANOVA, *p*<0.01), but there was no significant effect of 80 mM ethanol on the development of post-tetanic potentiation at the longer recovery intervals. **(B)** In BLA neurons from B6 mice injected with virus expressing shRNA against bMunc13-2, interval significantly also increased the expression of post-tetanic potentiation (two-way ANOVA, ^**^p < 0.01) but this was significantly suppressed by ethanol exposure (## *p* < 0.01). This effect was specifically significant at the 10 s interval where post-tetantic potentiation was maximal over this interval range (Bonferroni's multiple comparison post-test, ^*^p < 0.05).

On the other hand, in the absence of ethanol, the Munc13-2 shRNA significantly increased first response amplitude in the first train relative to the first response in the second train (Figure 3C_1_, green traces, two-way ANOVA comparing no ethanol condition with interval and knockdown as the main factors, interval—*p* < 0.001, knockdown—*p* < 0.05). This is consistent with previous findings showing greater vesicle recycling in mouse strains expressing lower Munc13-2 levels (Gioia et al., 2016; Gioia and McCool, 2017). More importantly, acute treatment with ethanol significantly slowed the RRP recovery in cells from Munc13-2 knockdown mice (Figure 3C_2_, two-Way ANOVA, main effects of recovery interval, *p* < 0.001, and ethanol *p* < 0.01, with Dunnett's *post-hoc* comparison finding a significant difference at the 10 s interval *p* < 0.05). These results are consistent with an effect of ethanol on inhibiting synaptic vesicle recycling after knockdown of Munc13-2 proteins in B6 mice. In addition to this AUC measure of the RRP, we also compared the amplitude of the first response in the first train to the first response in the second train. Like the AUC, the response amplitude in the second train went from a small fraction of the first response/first train amplitude at the 1 s inter-train interval to become much greater than the first response/first train amplitude by the 10 s inter-train interval (Figure 4). In animals injected with the scrambled control virus (Figure 4A), acute ethanol (80 mM) did not alter this development of PTP as inter-train intervals increased (two-way ANOVA, ethanol *p* = 0.90, interval *p* < 0.01). However, in animals expressing the bMunc13-2 shRNA (Figure 4B), acute ethanol significantly decreased the development of PTP with an especially prominent suppression of PTP at the 10 s interval (two-way ANOVA, main effect of ethanol *p* < 0.05 and interval *p* < 0.001, Bonferroni's post-test *p* < 0.05 at the 10 s interval). These findings indicate that the ethanol-dependent reduction in RRP recovery in bMunc13-2 knockdown animals also interrupts post-tetanic potentiation.

## Discussion

In these studies we confirmed our hypothesis that knockdown of Munc13-2 proteins increases the sensitivity of presynaptic terminals to ethanol mediated inhibition of synaptic vesicle recycling. Considering that Munc13-1 and Munc13-2 isoforms both contribute to glutamate release in the BLA (Gioia et al., 2016), these results suggest that vesicles primed by Munc13-1 are more sensitive to the inhibitory effects of alcohol than those primed by a combination of Munc13-1 and Munc13-2. These results may explain why B6 synapses, which are enriched in Munc13-2 relative to other strains (Gioia et al., 2016) are insensitive to the acute effects of alcohol on vesicle recycling. While Munc13-2 knockdown did not influence protein levels of Munc13-1 suggesting the shRNA was specific for the target protein, we did also find a down-regulation of protein levels for the Munc13-2 binding protein, RIM1α, and the SNARE complex protein, SNAP-25. RIM1α is part of the protein scaffold that regulates release probability and short-term plasticity (Schoch et al., 2002) while SNAP25 is an extensively-studied t-SNARE membrane protein that complexes with syntaxin and play a critical role in vesicle fusion. Notably, protein levels of Syntaxin1 (t-SNARE) an the v-SNARE VAMP1were not altered by the knockdown suggesting decreases in RIM1α and SNAP-25 protein levels were not part of any global down-regulation in release machinery function. Rather, these effects likely reflect a compensatory mechanism attempting to offset increased glutamate release and short-term plasticity following Munc13-2 knockdown (Gioia et al., 2016; and this work). Finally, considering both that stria terminalis inputs to the BLA undergo a presynaptic form of plasticity following chronic ethanol exposure (Christian et al., 2013) and that Munc13-1 proteins are important for long term presynaptic plasticity (Yang and Calakos, 2011), it is likely that Munc13-depdendent processes could alter the development of withdrawal-related synaptic physiology.

Notably, our data all reflect bilateral knockdown of Munc13-2. Lateralization of the lateral/basolateral amygdala is well-studied in human imaging studies; and there are many reviews across multiple sensory modalities. In general, they together suggest that bilateral amygdala activation can be associated with both positive and negative emotional processing. But, issues related to the differential activation time-course for left and right amygdala, temporal resolution of the imaging modality, stimulus complexity, habituation, and expectancy/context can all bias measured activation patterns toward one side or the other (Baas et al., 2004; Sergerie et al., 2008; Fusar-Poli et al., 2009; Patin and Pause, 2015). Translating laterization to rodent amygdala is therefore problematic and not well-represented in the murine literature. In rats where lateralization is better studied, context and the emotional valence of the stimulus again can shift amygdala activation to the right or left hemisphere. Studies employing negative affective training or exposures generally find increased expression of biochemical markers associated with neuron activity in the right lateral/basolateral amygdala while those using positive valence exposures find more marker activation in the left lateral/basolateral amygdala (Scicli et al., 2004; Alvarez and Banzan, 2011; Young and Williams, 2013). Future work focused on the positive and negative behavioral effects of ethanol and potential lateralized contributions of Munc13-2 in this context is certainly warranted.

### Ethanol modulation of synaptic vesicle recycling

At rest, synaptic vesicles are maintained in distinct vesicle pools dependent upon their interactions with various intracellular scaffolding proteins. Vesicles that are docked and primed at the presynaptic active zone are collectively referred to as the RRP, as only these vesicles are able to be released in response to action potentials. A second pool of vesicles, the recycling pool, is located nearby the active zone but not docked or primed for release. During repetitive stimulation, the RRP becomes depleted when the release demands exceed the refilling rate of the RRP, leading to synaptic failures (Neher and Sakaba, 2008). Therefore, the refilling or recycling rate of these vesicles determines the capacity of cells to maintain release during repetitive activation. In order to refill the RRP, vesicles from the recycling pool have to dock to the active zone and undergo an obligatory priming reaction which allows the vesicles to form stable SNARE complexes. This priming process is dependent on a specific domain that is present in all Munc13 isoforms termed the Munc Homology Domain (MHD). During vesicle priming, the MHD binds to Munc18-1/Syntaxin1 heterodimers and allows Syntaxin1 to participate in the formation of SNARE complexes (Ma et al., 2011).

Diacylglycerol (DAG) binds to the C1 domain of Munc13 proteins to facilitate vesicle priming (Betz et al., 1998; Rhee et al., 2002). It has previously been shown that ethanol binds to the Munc13-1 isoform within its C1 DAG-binding domain and is thought to displace DAG from its binding site (Das et al., 2013). Accordingly, this action would be predicted to inhibit vesicle priming rates. Therefore, we hypothesized that ethanol would disrupt synaptic vesicle recycling in a Munc13-1 dependent manner. Although it is currently unknown if ethanol binds to Munc13-2 isoforms, there is evidence for differential effects of DAG binding to Munc13-2 compared to Munc13-1 (Rosenmund et al., 2002). Additionally, Munc13-1 and Munc13-2 have highly divergent N-terminal regions (Betz et al., 2001), making it possible that the inhibitory effects of ethanol could be limited to Munc13-1 dependent priming without interfering with Munc13-2 dependent priming. Indeed, our results are congruent with this interpretation, such that the ratio of Munc13-1:Munc13-2 isoforms determines ethanol sensitivity. Therefore, by decreasing Munc13-2 levels with the shRNA we were able to remove the ethanol insensitive or “protected” mode of vesicle priming, thereby increasing the ethanol sensitivity.

### Ethanol modulation of post-tetanic potentiation

Post-tetanic potentiation (PTP) is a presynaptic form of short term plasticity that occurs in the seconds immediately following high frequency activity (Habets and Bost, 2005). This process is mechanistically similar to the processes involved in refilling of the RRP. For example, repetitive activation of presynaptic terminals leads to the accumulation of second messengers such as DAG and calcium/calmodulin within the presynaptic terminal which both aid in the refilling of the RRP through Munc13 dependent processes (Lipstein et al., 2013). After the cessation of stimulation these second messengers remain elevated in the terminal for several seconds and continue to add to the size of the RRP. A subsequent stimulation within the next couple of seconds accordingly produces a larger post-synaptic response due to increased number of vesicles being released (Habets and Bost, 2005). DAG and calcium/calmodulin bind independently to distinct Munc13 domains and dramatically enhance function. As with the priming events outlined above, DAG accumulation during repeated synaptic stimulation enhances membrane translocation Munc13-1 (Betz et al., 1998) which would both facilitate release rate (Basu et al., 2007) and vesicle maintenance (Rhee et al., 2002) during periods of high synaptic demand. While calcium/calmodulin binding to the conserved binding site in Munc13 proteins does not appear to impact basal neurotransmitter release, it strongly regulates short-term plasticity by enhancing the size of the readily releasable vesicle pool (Junge et al., 2004; Lipstein et al., 2012). However, mutations within the calcium/calmodulin binding site do not disrupt facilitation of synaptic transmission by phorbol esters (DAG analogs) suggesting that these 2 s messengers may act independently to regulate short-term plasticity. Considering that ethanol inhibits vesicle recycling through some of the same mechanisms involved in PTP, we predicted that ethanol would reduce the size of PTP in a Munc13-1 dependent manner. Indeed, we found that ethanol reduced the magnitude of PTP specifically in Munc13-2 KD cells. This suggests that Munc13-2 isoforms may simply be insensitive to the inhibitory effects of ethanol on PTP. Alternatively, repetitive stimulation of synapses expressing only Munc13-1 produces a modest short-term augmentation while short term-augmentation is much more robust in synapses expressing only Munc13-2 (Junge et al., 2004). Acute ethanol inhibition of vesicle recycling and subsequently post-tetanic plasticity may thus be more apparent when short-term augmentation is reduced by reducing Munc13-2 levels. Regardless of the mechanism, our results strongly suggest the relative ratio of Munc13-1:Munc13-2 dependent priming regulates the ethanol sensitivity of short-term post-tetanic plasticity.

### Behavioral consequences

What might the behavioral consequences be for ethanol modulation of synaptic release during periods of robust synaptic activation? Although the ethanol concentration-response relationship suggests (Figure 2) suggests a moderate potency on vesicle recycling in our *ex vivo* experiments, high frequency activity within prefrontal cortical neuronal ensembles is associated with a number of behaviors related to executive function and cognitive flexibility. These ensembles often produce action potentials in the 10–30 Hz range for several hundred milliseconds (Meyers et al., 2012; Simon et al., 2015). This suggests that exposure to intoxicating ethanol concentrations during these periods of intense activity may significantly impair the ability of prefrontal terminals to communicate with downstream brain regions via disruption in synaptic vesicle recycling. Such deleterious effects would potentially provide a neurobiological mechanism for ethanol disruption of executive function and decision making (Weissenborn and Duka, 2003; Montgomery et al., 2011; Weafer and Fillmore, 2015). With respect to mPFC terminals in the BLA, fear conditioning involves presynaptic plasticity in later region; and this conditioning is altered in animals that are chronically exposed to ethanol (Holmes et al., 2012). Interestingly, during withdrawal from ethanol exposure, D2 mice exhibit facilitated freezing in response to fear associated cues (Tipps et al., 2015). Considering that D2 mice express a higher ratio of Munc13-1 to Munc13-2 expression in the BLA than B6 mice (Gioia et al., 2016) this may suggest that ethanol effects through Munc13-1 produce a vulnerable state at BLA synapses which subsequently facilitates the presynaptic plasticity associated with fear learning.

## Summary

In conclusion, ethanol interferes with presynaptic signaling likely through interactions with Munc13-1 proteins. This acute effect of ethanol inhibits the ability of cells to maintain release during high levels of demand. Considering that cells often increase their firing rate in response to aversive environments in a manner that is correlated with the expression of anxiety-like behavior (Wang et al., 2011), it is likely that alcohol effects through Munc13 proteins may contribute to the anxiolytic and cognitive dysfunctional effects of alcohol.

## Ethics statement

This study was carried out in accordance with the recommendations of US Public Health Service Policy on Humane Care and Use of Laboratory Animals. The protocol was approved by the Wake Forest Institutional Animal Care and Use Committee.

## Author contributions

DG performed the experiments, performed data analysis, and wrote initial drafts of the manuscript. NA performed the western analysis. BM supervised experimental execution, performed data analysis, made substantial contributions for experimental design, and wrote the final submitted form of the manuscript. All authors agree to be accountable for all aspects of the work.

### Conflict of interest statement

The authors declare that the research was conducted in the absence of any commercial or financial relationships that could be construed as a potential conflict of interest.
